# Facile Synthesis of Cross-linked Hyperbranched Polyamidoamines Dendrimers for Efficient Hg(Ⅱ) Removal From Water

**DOI:** 10.3389/fchem.2021.743429

**Published:** 2021-09-14

**Authors:** Xue Geng, Rongjun Qu, Xiangyu Kong, Shengnan Geng, Ying Zhang, Changmei Sun, Chunnuan Ji

**Affiliations:** ^1^ School of Chemistry and Materials Science, Ludong University, Yantai, China; ^2^ Yantai Research Institute for the Transformation of Old and New Kinetic Forces, Yantai, China

**Keywords:** hyperbranched polyamidoamine dendrimer, crosslinking, epichlorohydrin, ethylene glycol diglycidyl ether, Hg(Ⅱ), adsorption

## Abstract

Dendrimers as commonly used metal ions adsorption materials have the advantages of good adsorption performance and high reuse rate, but the high cost limits its extensive use. Compared with dendrimers, hyperbranched dendrimers have similar physical and chemical properties and are more economical. Therefore, hyperbranched dendrimers are more suitable for industrial large-scale adsorption. The hyperbranched polyamidoamine (HPAMAM) gels were prepared by cross-linking hyperbranched polyamidoamine (HPAMAM-ECH-x and HPAMAM-EGDE-x) with different amounts of epichlorohydrin (ECH) and ethylene glycol diglycidyl ether (EGDE), respectively. The as-synthesized adsorbents were characterized by FT-IR, SEM and XPS. The prepared adsorbents were used to adsorb Hg(Ⅱ) in aqueous solution, and the effects of solution pH, contact time, temperature and initial concentration of metal ion on the adsorption capacity were investigated. The effect of solution pH indicated that the optimum condition to Hg(Ⅱ) removing was at pH 5.0. The adsorption kinetic curves of the two kinds of materials were in accordance with the pseudo-second-order model. For the HPAMAM-ECH samples, the adsorption thermodynamic curves fitted the Langmuir model, while for the HPAMAM-EGDE samples, both Langmuir and Freundlich equations fitted well. The maximum adsorption capacity of HPAMAM-ECH-3 obtained from Langmuir model toward Hg(Ⅱ) was 3.36 mmol/g at pH 5.0 and 35°C.

## Introduction

Pollution prevention and control has always been a serious problem with social development. Heavy metal ions such as Hg(Ⅱ), Cu(Ⅱ), Cd(Ⅱ) and Pb(Ⅱ) are one of the most discharged pollutants, which cause great harm to water body, soil and human health (Zamora-Ledezma et al., 2021). Among them, Hg(Ⅱ) is considered highly toxic because it can do so much damage to organisms at trace levels (Bao et al., 2021). Hg(Ⅱ) pollution mainly comes from the mining industry, battery production, electroplating and textile industries (Fan et al., 2019). When it is discharged into water, it will not only be absorbed by aquatic plants and animals, but also infiltrate into the soil and affect crops, ultimately causing great harm to human health (Li et al., 2019). Therefore, various methods have been used to remove Hg(Ⅱ) from wastewater (Ding et al., 2018; Lan et al., 2021). Adsorption method is considered to be the most promising water treatment method, because it has the advantages of simple operation, low energy consumption, no secondary pollution and other advantages. At the same time, it has the characteristics of easy recycling of product removal, in line with the requirements of circular economy and sustainable development (Liu et al., 2018; Hu et al., 2021). Adsorbents containing nitrogen and oxygen atoms are commonly used as one of the most effective adsorbents (Liao et al., 2021a; Liao et al., 2021b; Zhuang et al., 2021). Over the past few decades, a variety of adsorbent materials have been prepared to remove mercury ion contamination from wastewater, such as cellulose (Velempini et al., 2019), magnetic carbon nanotubes (Fayazi, 2020), dendritic polymers (Ma et al., 2019), modified fibers (Xu et al., 2016), chitosan (Hou et al., 2018), etc.

Polyamidoamine (PAMAM) dendrimer stands out among many adsorbents because of their unique and excellent properties. The PAMAM dendrimer is characterized by a highly branched monodisperse three-dimensional structure, which gives it high surface activity and versatility (Niu et al., 2014). As an adsorption material, PAMAM dendrimer contains a large number of nitrogen and oxygen atoms that can adsorb metal ions (Ren et al., 2019). Meanwhile, the existence of cavity inside the molecule can accommodate metal ions, which makes it have the advantage of high adsorption capacity (Yin et al., 2019). In addition, PAMAM dendrimer can also regulate and control the adsorption capacity and adsorption selectivity to the adsorbate, because its relative molecular weight can be controlled by the number of branch iterations. There have been many reports on removal of metal ions from wastewater by PAMAM dendrimer. Qiao et al. prepared Schiff base functionalized polyamidoamine dendrimers/silica as adsorbent by grafting salicylaldehyde onto the polyamides dendrimers/silica, and investigated the adsorption behavior for Co(II) and Mn(II). The results indicated that the synthesized adsorbent exhibited high adsorption capacities and adsorption efficiency to Co(II) and Mn(II) (Qiao et al., 2020). Ma et al. synthesized different generations of polyamidoamine dendrimers functionalized magnetic graphene oxide *via* step-by-step growth chemical grafting approach and magnetic separation technology. The results showed that the composite adsorption material had a good adsorption property for Hg(Ⅱ) and the Hg(Ⅱ) was reduced to Hg(Ⅰ) during the adsorption process (Ma et al., 2017).

However, the synthesis of PAMAM dendrimer requires tedious and complex multistep reactions, each of which requires strict protection and separation and purification measures (Inoue, 2000). Although researchers are working to reduce steps required to synthesize PAMAM dendrimer, the high cost due to stepwise synthesis mechanism has limited its widespread use. At the same time, in many applications where perfect structures are not required, hyperbranched polyamidoamine dendrimer (HPAMAM) emerges (Seiler et al., 2004). The HPAMAM dendrimer is very much like PAMAM dendrimer with a similar structure and properties (Lin et al., 2018). Compared with the PAMAM dendrimer, the HPAMAM dendrimer can be synthesized by one step reaction with suitable AB_2_ monomer, the molecule is irregular ellipsoidal and the molecular weight is polydispersive (Saadati et al., 2021). The one-pot synthesis technology of HPAMAM dendrimer has advantages of low production cost and short production cycle, making it economically acceptable for large-scale industrial applications. In recent years, researchers tend to use hyperbranched polyamidoamine dendrimer instead of polyamidoamine dendrimer to prepare adsorption materials (Wang et al., 2014). However, there is a problem that cannot be ignored is that HPAMAM, like PAMAM, is also soluble, and it is difficult to separate HPAMAM when used as adsorption material directly. Therefore, researchers often immobilize it on a variety of substrates to remove heavy metal ions from wastewater (Zhu et al., 2018). Wang et al. prepared hyperbranched polyamide functionalized sodium alginate microspheres by cross-linking with glutaraldehyde and investigated their selective adsorption performances and reusability to Sb(Ⅲ). The results showed that the synthesized adsorbent exhibited favorable adsorption capacity and selectivity for Sb(Ⅲ). After eight adsorption-desorption, the adsorption capacity of microspheres retained more than 90%, and the selectivity was relatively stable (Wang et al., 2020). In our previous work (Zhang et al., 2019), we synthesized triethylenetetramine terminal hyperbranched dendrimer modified silica gel adsorbents using long-chain TETA as a reaction unit through the combination of homogeneous method and heterogeneous method. The resulting composites exhibited more active sites and flexibility, and thus can remove Au(Ⅲ) more efficiently. The adsorption of the SG-TETA2 rapidly reached equilibrium within 90 min, while for the SG-TETA equilibrium was reached after 120 min.

In the present study, our aim is to find a simple and rapid method to prepare an economical and efficient hyperbranched polyamide adsorption material, which has a favorable adsorption capacity and selectivity. Two kinds of HPAMAM adsorbents were prepared by cross-linking soluble hyperbranched polyamides with epichlorohydrin (ECH) and ethylene glycol diglycidyl ether (EGDE) respectively. The effects of the type and amount of crosslinking agent on the morphology and structure of the materials that we prepared in this work and on the adsorption properties for Hg(II) were studied by a series of adsorption experiments including static saturation, optimum pH, adsorption isotherms, adsorption kinetics, adsorption selectivity, as well as adsorption mechanism.

## Experimental

### Materials

HPAMAM were provided by Weihai CY Dendrimer Technology Co., Ltd., China. Epichlorohydrin was bought from Sinopharm Chemical Reagent Co., Ltd., China. Ethylene glycol diglycidyl ether was purchased from Shanghai Macklin Biochemical Co., Ltd., China. Mercuric nitrate (Hg(NO_3_)_2_⋅H_2_O) was purchased from Shanghai Aladdin Biochemical Technology Co., Ltd., China. All of the other reagents were analytically pure and used as received without further purification.

### Instruments and Apparatus

Infrared (IR) spectra were measured on a Fourier transform infrared (FTIR) spectrophotometer Nicolet iS50 (Nicolet, American). Surface morphologies were examined using Field Emission Scanning Electron Microscope (FE-SEM SU8010) (Hitachi, Japan). X-ray photoelectron spectroscopy (XPS) was performed on ESCALAB Xi^+^ (Thermo Fisher Scientific, American). Elemental analysis was obtained from the Elementar VarioEL III instrument, Elementar Co., Germany. The parameters of the porous structures were determined using an automatic physisorption analyzer (ASAP 2020; Micromeritics, United States).

### Preparation of HPAMAM-ECH, and HPAMAM-EGDE

**SCHEME 1:**
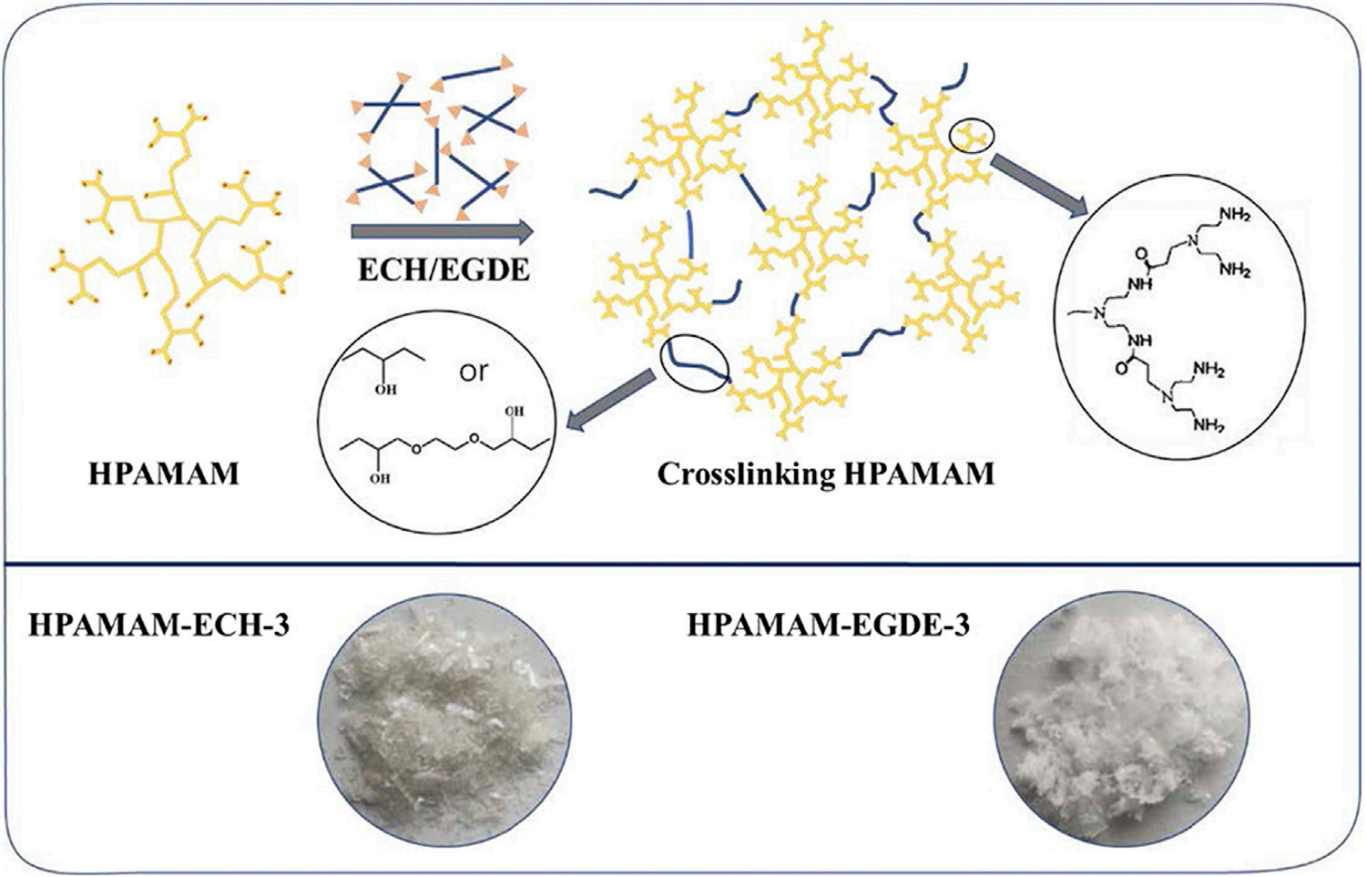
Schematic diagram of the crosslinking process.

Different numbers of epoxy groups, such as epoxy chloropropane (ECH, single epoxy group) and ethylene glycol diglycidyl ether (EGDE, diepoxy group) were selected as cross-linkers. The cross-linking of HPAMAM was carried out by adding a specific amount of the cross-linker into a methanolic solution of HPAMAM, in which the concentration of HPAMAM was 20 g/L. The molar ratio of HPAMAM and the cross-linker (ECH or EGDE) varied from 1:2 to 1:5. The cross-linking reaction was performed at 60°C for 12 h under magnetic stirring and then the solvent was removed in a drying oven at 80°C for 24 h. After the solvent has evaporated completely, the resulting colorless transparent gel was extracted with refluxing ethanol in a Soxhlet extractor for 12 h and dried under vacuum at 80°C for 6 h. Here, the resultants were notated as HPAMAM-ECH (or EGDE)-x, where x designated molar ratio of epoxy crosslinker to HPAMAM. The specific dosages of each product were shown in Table 1 and the synthetic process of cross-linked hyperbranched polyamidoamine illustrated in Scheme 1. It can be seen from the Scheme 1 that the product obtained by ECH cross-linking were flaky solid, while the products obtained by EGDE cross-linking were soft and loose floccule.

**TABLE 1A:** Formulation of HPAMAM-ECH samples.

Molar ratio of HPAMAM/ECH	Dosage of HPAMAM (g)	Dosage of ECH (ml)	Product
HPAMAM/ECH = 1:2	2.0	0.48	HPAMAM-ECH-2
HPAMAM/ECH = 1:3	2.0	0.72	HPAMAM-ECH-3
HPAMAM/ECH = 1:4	2.0	0.96	HPAMAM-ECH-4
HPAMAM/ECH = 1:5	2.0	1.20	HPAMAM-ECH-5

**TABLE 1B:** Formulation of HPAMAM-EGDE samples.

Molar ratio of HPAMAM/EGDE	Dosage of HPAMAM (g)	Dosage of EGDE (ml)	Product
HPAMAM/EGDE = 1:2	2.0	0.42	HPAMAM-EGDE-2
HPAMAM/EGDE = 1:3	2.0	0.63	HPAMAM-EGDE-3
HPAMAM/EGDE = 1:4	2.0	0.84	HPAMAM-EGDE-4
HPAMAM/EGDE = 1:5	2.0	1.05	HPAMAM-EGDE-5

### Adsorption Experiments

The adsorption experiments were carried out as follows: the adsorbing material (10 mg) was added to the solution (40 ml) of Hg(NO_3_)_2_ of different concentrations. The mixture was then shaken in thermostatic oscillating chamber for 24 h at pH 5.0 and 25°C, the pH value was adjusted with dilute nitric acid (HNO_3_), or aqueous sodium hydroxide (NaOH). The original and final concentrations of the metal ion were measured by atomic absorption spectrometry (AAS). The adsorption capacity was then calculated as follows:

$$q_{e}=\frac{\left(C_{0}-C_{e}\right)V}{W}$$ (1)

Where *q*_e_ (mmol⋅g^−1^) is the equilibrium adsorption capacity of the composite, *C*_*0*_ (mmol⋅L^−1^) and *C*_*e*_ (mmol⋅L^−1^) represent the initial and equilibrium concentration of metal ion, respectively, *V* (L) is the volume of the metal solution, and *W* (g) is the weight of the adsorbent.

The effect of pH on the uptake of adsorbents for Hg(II) was assessed, and the range of pH was chosen from 1.0 to 6.0. The adsorption kinetics of the adsorbent for Hg(II) was investigated by measuring concentrations of Hg(II) in the solution at regular time intervals from 0 to 12 h. In addition, the effect of Hg(II) initial concentration at pH 5.0 and 15, 25, and 35°C was also studied. The initial concentrations were 50, 100, 150, 200, 250 and 300 mg/L, respectively.

The adsorption selectivity of HPAMAM-ECH (or EGDE)-x was established by analyzing a series of binary metal ion systems with Hg(II) and a coexisting metal ion including Cu(II), Pb(II), Cd(II), Ni(II) and Zn(II). The adsorption selective coefficient (α) was calculated by the equation as follows:

$$\alpha \;=\frac{The\;adsorption\;capacity\;of\;Hg\left(II\right)\;on\;adsorbent\left(mmol/g\right)}{The\;adsorption\;capacity\;of\;coexisting\;metal\;ion\;on\;adsorbent\left(mmol/g\right)}\;$$ (2)

The regeneration performance of adsorbents was evaluated by using 0.1 mol⋅L^−1^ HNO_3_-3% thiourea as the eluent, the adsorbed materials (10 mg) was added to the eluent (40 ml) and shaken at 25°C for 24 h, then the concentration of Hg(II) in the eluent was determined. The ratio of the concentration of Hg(II) in the eluent to the concentration of Hg(II) in the adsorbed material was the regeneration rate. The recovered adsorbents were once again added to a solution (40 ml) of 100 mg/L Hg(NO_3_)_2_ at pH 5.0, shaken at 25°C for 24 h and the adsorption volume was calculated. The elution-regeneration experiment was repeated for three times.

## Results and Discussion

### Characterization of Adsorbents

#### IR Spectroscopy Analysis

**FIGURE 1:**
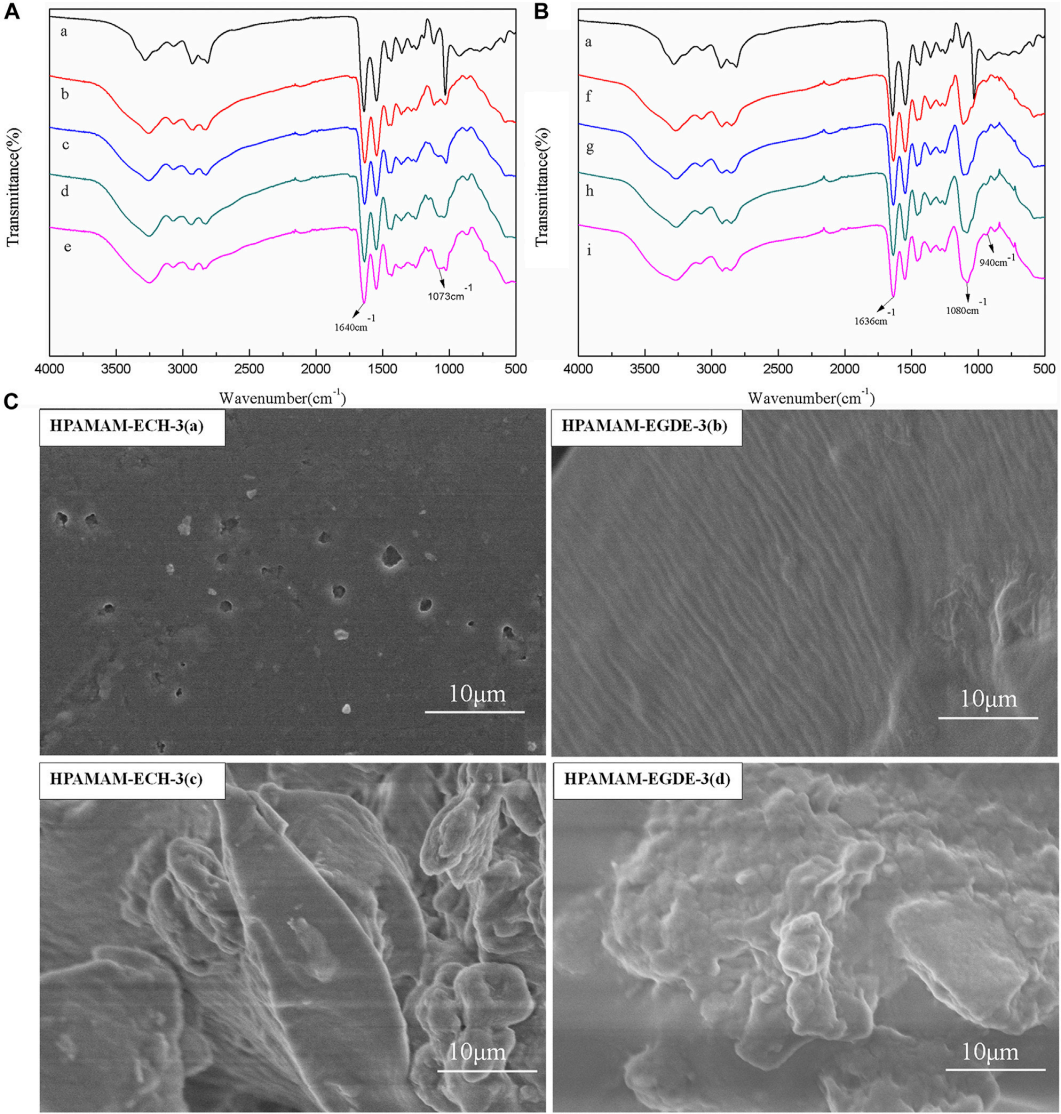
**(A)** IR spectra of HPAMAM and HPAMAM-ECH samples: (a) HPAMAM, (b) HPAMAM-ECH-2, (c) HPAMAM-ECH-3, (d) HPAMAM-ECH-4, (e) HPAMAM-ECH-5; **(B)** IR spectra of HPAMAM and HPAMAM-EGDE samples: (a) HPAMAM, (f) HPAMAM-EGDE-2, (g) HPAMAM-EGDE-3, (h) HPAMAM-EGDE-4, (i) HPAMAM-EGDE-5; **(C)** FE-SEM images of HPAMAM-ECH-3 and HPAMAM-EGDE-3: (a, b) surface morphology, (c, d) internal form.

The FT-IR spectra of HPAMAM-ECH and HPAMAM-EGDE were showed in Figure 1A and Figure 1B, respectively. It can be observed that the absorption peaks around 3284 and 1640 cm^−1^ in HPAMAM, which correspond to the N-H stretching vibration of amino and C=O stretching vibration of amide (Oliveira et al., 2021), respectively. After crosslinking with ECH, the stretching vibration peak of C=O were weakened and the C-O-C stretching vibration appeared at 1073 cm^−1^ (Abdulhameed et al., 2019), which indicated the successful crosslinking between HPAMAM and ECH. The same phenomenon was observed in samples of HPAMAM-EGDE, the new peaks appeared around 1080 cm^−1^ were assigned to the C-O-C stretching vibration in EGDE. In addition, the peak at 940 cm^−1^ attributed to the symmetric stretching vibration absorption of epoxy functional group (Wang et al., 2017) was present in HPAMAM-EGDE but absent in all HPAMAM-ECH samples. This can be explained by the fact that a part of the epoxy groups in EGDE were not involved in the crosslinking reaction.

#### FE-SEM Images

The surface morphologies (Figure 1C) of the materials were determined by SEM *via* choosing HPAMAM-ECH-3 and HPAMAM-EGDE-3 as models. The surface of the material crosslinked with epichlorohydrin was relatively smooth, and the particles were irregularly shaped and tightly arranged. However, the surface of the material crosslinked with ethylene glycol diglycidyl ether showed a wrinkled structure with a row of grooves and the particle size was relatively large. The possible reason was that the molecular chain of ethylene glycol diglycidyl ether was longer than that of epichlorohydrin, and the spatial structure formed in the cross-linking process was looser.

#### BET Analysis

Specific surface area and pore size structure are important factors affecting the adsorption properties of materials. The BET results (Table 2) showed that the samples crosslinked with EGDE had relatively larger specific surface area and pore diameter than those crosslinked with ECH, which relying on nice stretch and flexibility of long-chain EGDE.

**TABLE 2:** The porous structures parameters of HPAMAM-ECH-3 and HPAMAM-EGDE-3.

Materials	BET surface area (m^2^/g)	BJH desorption cumulative volume of pores (cm^3^/g)	BJH desorption average pore diameter (nm)
HPAMAM-ECH-3	-	-	-
HPAMAM-EGDE-3	0.10	0.0004	12.37

#### XPS Analysis

**FIGURE 2:**
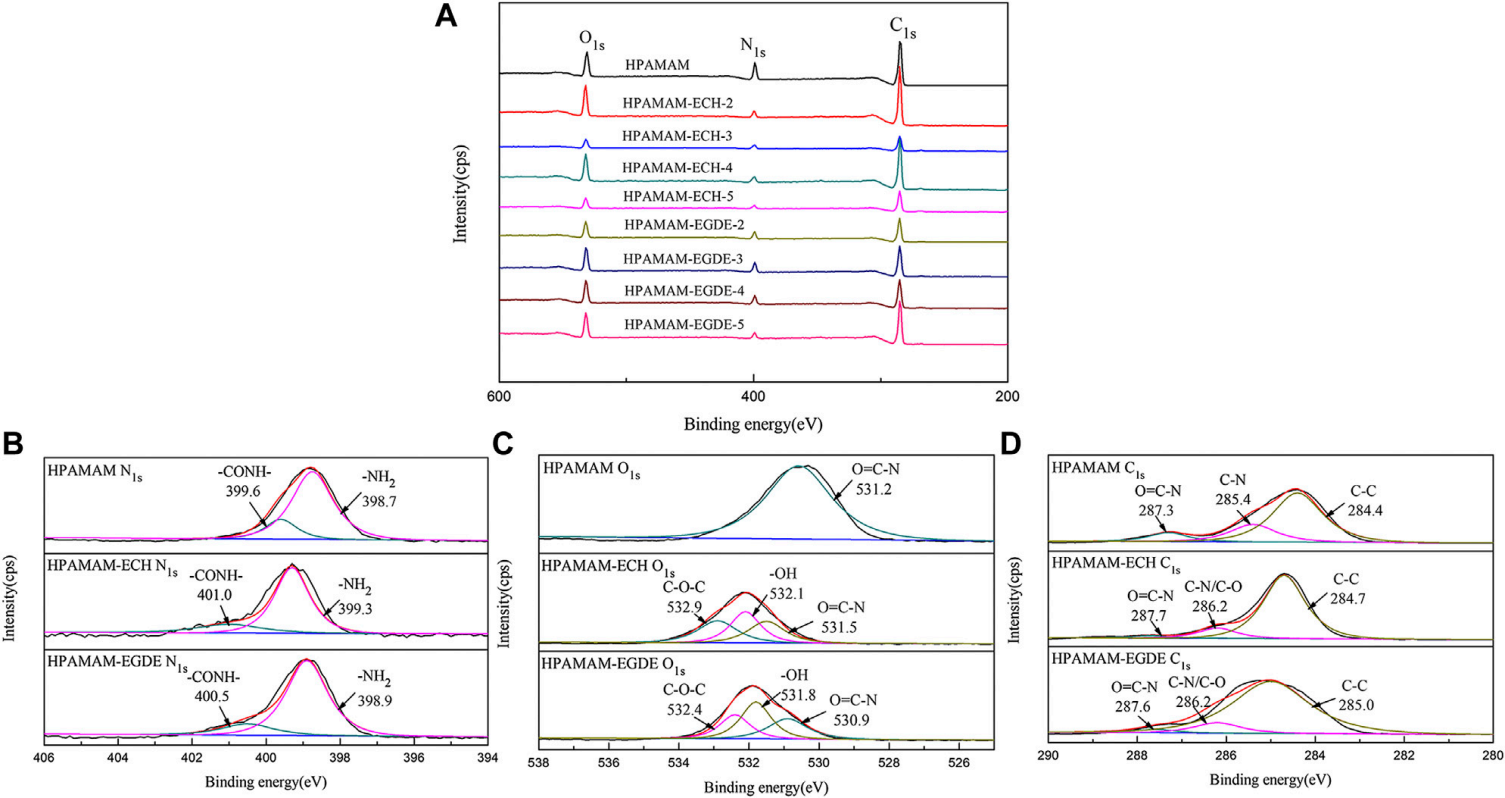
Wide scan XPS spectra of **(A)** HPAMAM-ECH samples and HPAMAM-EGDE samples, high-resolution XPS spectra of **(B)** N_1s_, **(C)** O_1s_, and **(D)** C_1s_.

XPS results of wide-scan spectra of HPAMAM-ECH samples and HPAMAM-EGDE samples were shown in Figure 2. In the figure of wide scan XPS spectra (Figure 2A), three peaks appeared at 532.1, 399.3 and 284.7 eV in all samples, which can be assigned to O_1s_, N_1s_ and C_1s_, respectively. What’s more, the ratios of O to N in both HPAMAM-ECH and HPAMAM-EGDE materials increased with the increase of the amount of crosslinking agent and were all greater than that in HPAMAM (Table 3). The high-resolution N_1s_ spectra (Figure 2B) of HPAMAM curve fitted to two peak components with bonding energies at 399.6 eV for the amide group, and at 398.7 eV for the amino group. After crosslinking, the bond energies of the two functional groups increased. O_1s_ (Figure 2C) in HPAMMA contained only one peak which belonged to amide group, while cross-linked HPAMAM contained three peaks. Three peaks of O_1s_ in HPAMAM-ECH samples: 532.9, 532.1 and 531.5 eV, corresponding to C-O-C group, -OH group and O=C-N group, respectively (Li et al., 2018). In the HPAMAM-EGDE sample, the bond energies corresponding to these three peaks were 532.4, 531.8 and 530.9 eV. The binding energy of C_1s_ (Figure 2D) in cross-linked HPAMAM also shifted to the left compared with HPAMMA. The binding energies of C_1s_ that appeared at about 288, 287 and 285 eV indicated there were three types of C in the crosslinking HPAMAM structure, which could be attributed to O=C-N group, C-N or C-O group and C-C group, respectively (Lin et al., 2018). The appearance of C-O group and -OH group was indicative of the presence of epoxy crosslinking agent in the materials, which was consistent with the results of IR spectroscopy analysis.

**TABLE 3:** Element concentrations of HPAMAM-ECH samples and HPAMAM-EGDE samples.

Samples	Element concentrations Atomic %				Functional groups content (mmol/g)
	C_1S_	N_1S_	O_1S_	O/N	NH_2_+CONH
HPAMAM	70.58	14.71	14.71	1.00	
HPAMAM/ECH = 1:2	70.33	13.23	16.44	1.24	8.07
HPAMAM/ECH = 1:3	75.43	10.13	14.44	1.43	7.39
HPAMAM/ECH = 1:4	76.61	6.42	16.97	2.64	6.92
HPAMAM/ECH = 1:5	77.18	6.14	16.68	2.72	6.25
HPAMAM/EGDE = 1:2	69.29	12.55	18.16	1.45	7.41
HPAMAM/EGDE = 1:3	67.84	12.83	19.31	1.51	6.56
HPAMAM/EGDE = 1:4	67.32	12.34	20.34	1.65	6.13
HPAMAM/EGDE = 1:5	76.65	6.57	16.78	2.55	5.32

### Adsorption Properties

#### Static Saturation Adsorption

**FIGURE 3:**
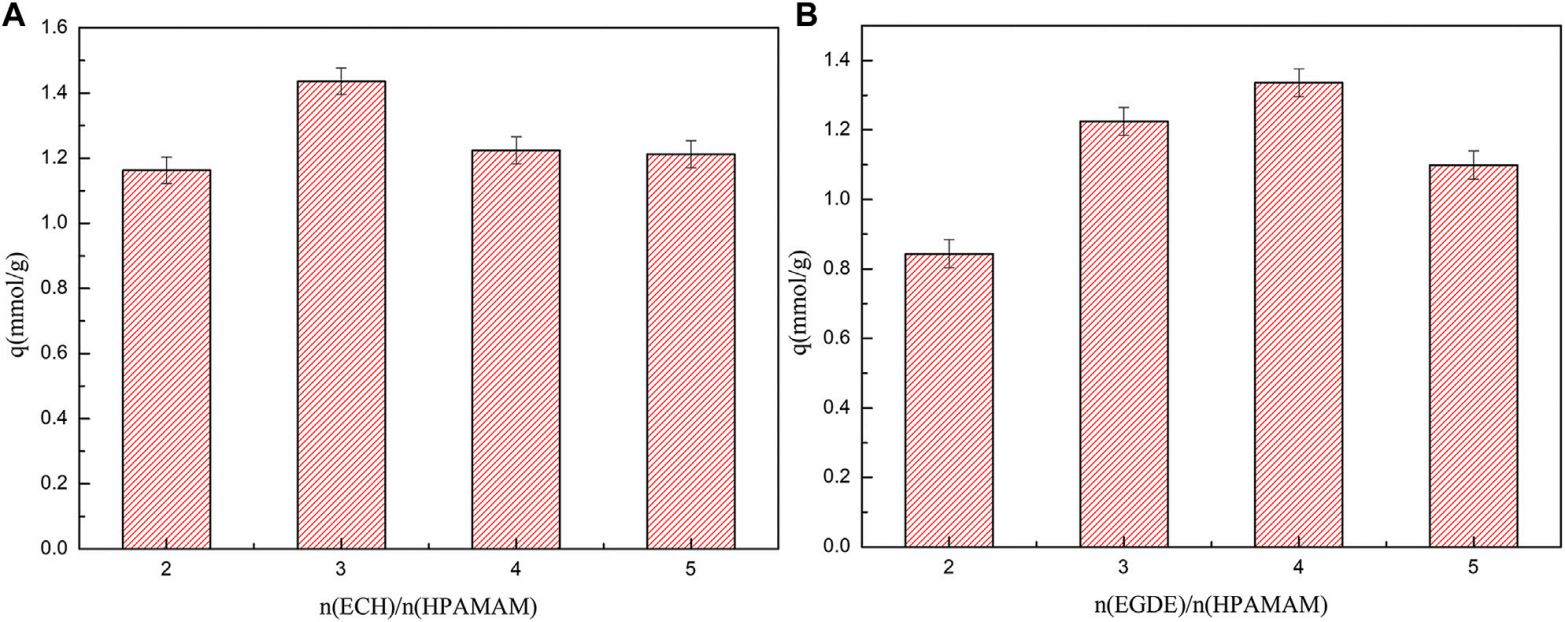
Saturated adsorption capacities for Hg(II) onto **(A)** HPAMAM-ECH samples and **(B)** HPAMAM-EGDE samples at pH 5.0, 25°C.

The saturated adsorption capacities of HPAMAM-ECH and HPAMAM-EGDE for Hg(II) were presented in Figure 3. For HPAMAM-ECH obtained using different quantity of ECH, the adsorption capacities for Hg(II) fell in order of HPAMAM-ECH-3 > HPAMAM-ECH-4 > HPAMAM-ECH-5 > HPAMAM-ECH-2, while in the case of HPAMA-EGDE, the order was HPAMAM-EGDE-4 > HPAMAM-EGDE-3 > HPAMAM-EGDE-5 > HPAMAM-EGDE-2. This phenomenon can be explained as the structure and morphology of the material changed with the increase of the amount of crosslinking agent, resulting in the increase of the adsorption capacity. However, with the further increase of the amount of crosslinking agent, the number of amino groups decreased, resulting in the decrease of the adsorption capacity.

#### Determination of the Optimum pH Value

**FIGURE 4:**
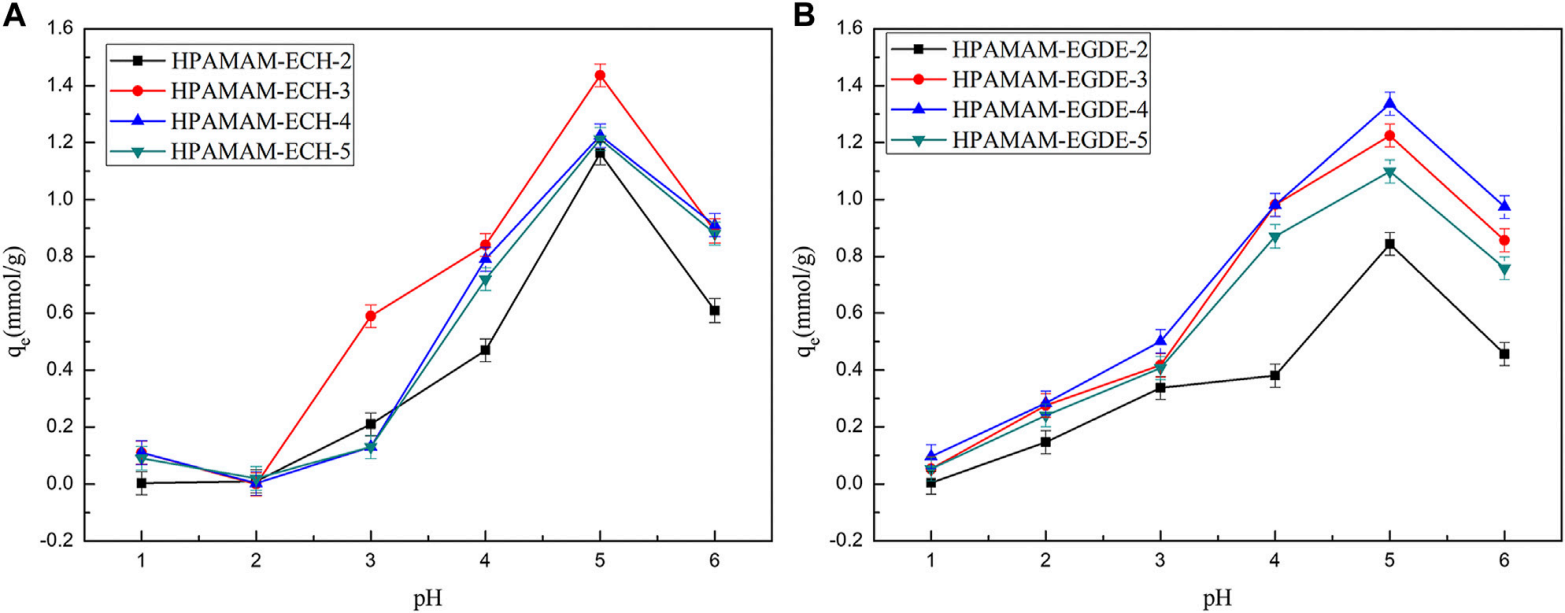
Effect of pH on the adsorption for Hg(II): **(A)** HPAMAM-ECH samples and **(B)** HPAMAM-EGDE samples.

The pH value of the solution is an important factor affecting the adsorption capacity of the adsorbent because it has a great influence on the state of the existence of metal ions and the state of the functional group of the adsorbent. Figure 4 showed that the adsorption capacities of the HPAMAM-ECH samples and HPAMAM-EGDE samples for Hg(II) responded differently to changing pH value. As the solution pH was varied from 1.0 to 6.0, the adsorbed quantity increased first and attained the maximum at pH 5.0 both for HPAMAM-ECH samples and HPAMAM-EGDE samples. At low pH, NH_2_ on the surface of adsorbents were positively charged owing to protonation, thus the adsorption was impeded by a large repulsive forces between the protonated amino group Hg(II). Moreover, the existence of a large amount of H^+^ in the solution may compete adsorption with Hg(II) (Zhang et al., 2021). The deprotonation of the NH_2_ on adsorbents can effectively coordinate with Hg(II), resulting in increased uptake of Hg(II) as pH increased from 1.0 to 5.0. Above pH 5.0, the adsorption capacities decreased with the increasing of pH. This could attributed to the formation of hydroxyl species of mercury such as soluble Hg(OH)^+^ (Zeng et al., 2021).

#### Adsorption Kinetics

**FIGURE 5:**
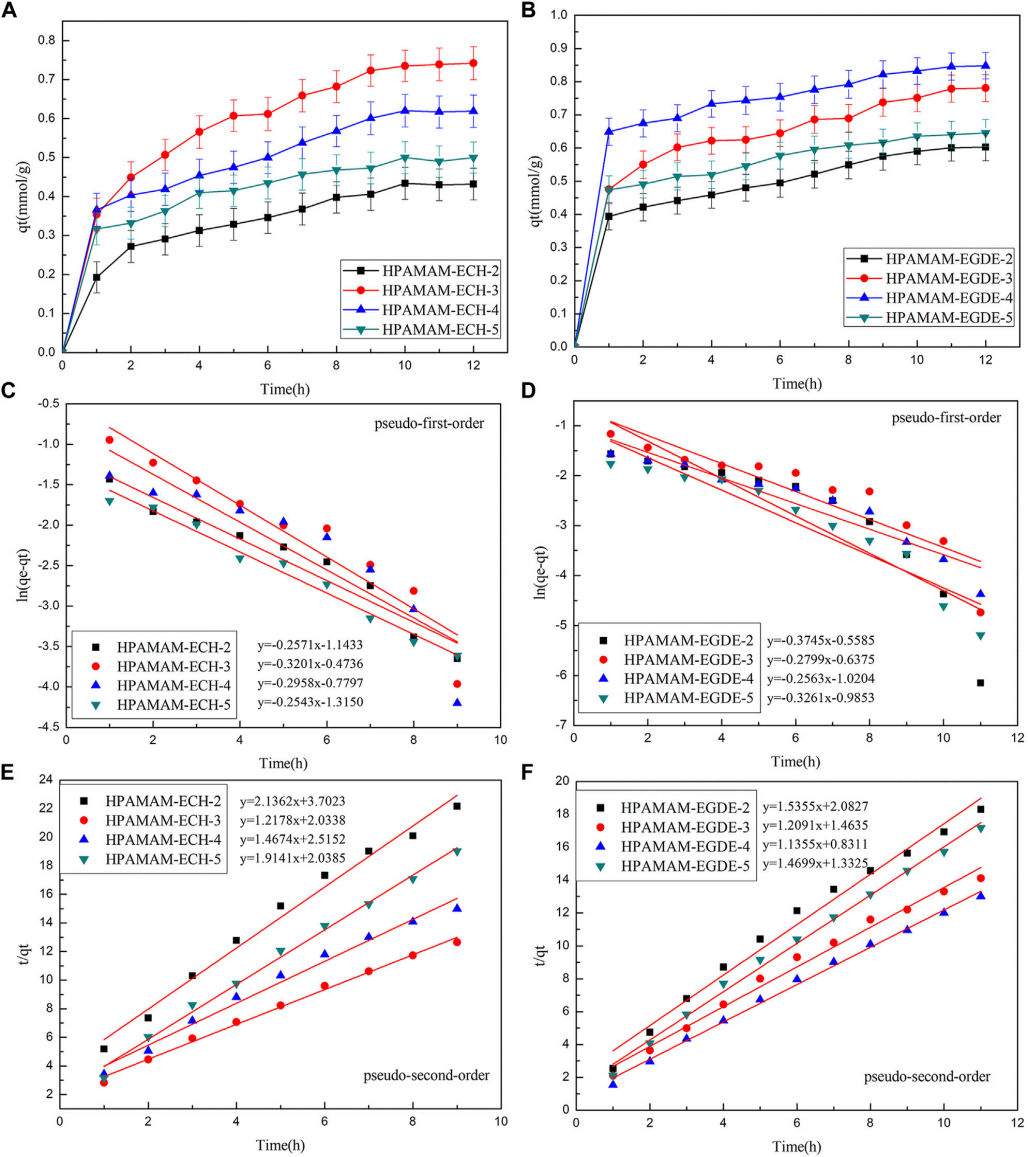
**(A**,**B)** Adsorption kinetics curves at 25°C, pH 5.0 of HPAMAM-ECH samples and HPAMAM-EGDE samples for Hg(II), **(C**,**D)** pseudo-first-order and **(E**,**F)** pseudo-second-order adsorption model.

Figure 5 showed the adsorption kinetic for Hg(II). The equilibrium kinetic adsorption capacity also followed the order of HPAMAM-ECH-3 > HPAMAM-ECH-4 > HPAMAM-ECH-5 > HPAMAM-ECH-2 for samples of HPAMAM-ECH and HPAMAM-EGDE-4 > HPAMAM-EGDE-3 > HPAMAM-EGDE-5 > HPAMAM-EGDE-2 for samples of HPAMAM-EGDE. For HPAMAM-ECH materials, the adsorption was swift at the inital 2 h and then tapered off until equilibrium was reached at about 10 h. Similarly, the adsorption within the first hour was rapid for HPAMAM-EGDE materials, which contributed most to the equilibrium adsorption and reached equilibrium at 10 h. The superiority of adsorption rate in the initial stage of HPAMAM-EGDE samples may come from the grooves of material surface and comparatively large surface area. The kinetics data were fitted into the pseudo-first-order and pseudo-second-order models (Niu et al., 2014):

$$ln\left(q_{e}\;-\;q_{t}\right)\;=\;lnq_{e}\;-\;k_{1}t\;$$ (3)

$$\frac{t}{q_{t}}=\frac{1}{k_{2}q_{e}^{2}}+\frac{t}{q_{e}}$$ (4)

Where *q*_*e*_ and *q*_*t*_ (mmol/g) are the adsorption capacity for Hg(II) at equilibrium and time *t* (h), and *k*_*1*_ and *k*_*2*_ are the rate constant of pseudo-first-order (h^−1^) and pseudo-second-order (g⋅mmol^−1^h^−1^) adsorption, respectively. Figure 5 and Table 4 gave fitting results and the kinetic parameters. The adsorption kinetic for the two materials fitted Pseudo-second-order model well as indicated by the higher correlation coefficients (R2 2), and the theoretical adsorption capacity was closer to the experimental value. Therefore, the adsorption kinetics of Hg(II) onto the adsorbents was better described by pseudo-second-order model. That is, the Hg(II) adsorbed rate determining step on HPAMMA-ECH and HPAMAM-EGDE may be dominant by chemisorption but not mass transfer in solution (Qu et al., 2018).

**TABLE 4:** Kinetic parameters for the adsorption of Hg(II) on all samples.

Adsorbents	*q*_e, exp_ (mmol/g)	Pseudo-first-order kinetics			Pseudo-second-order kinetics		
		*K*_*1*_(h^−1^)	*q*_*e,*__cal 1_ (mmol/g)	R^2^_1_	*K*_2_ (g/mmol h)	*q*_*e,*__cal 2_ (mmol/g)	R^2^_2_
HPAMAM-ECH-2	0.434	0.257	0.319	0.9396	1.233	0.468	0.9849
HPAMAM-ECH-3	0.742	0.320	0.623	0.8926	0.729	0.821	0.9940
HPAMAM-ECH-4	0.620	0.296	0.459	0.8016	0.856	0.682	0.9846
HPAMAM-ECH-5	0.500	0.254	0.269	0.9798	1.798	0.522	0.9933
HPAMAM-EGDE-2	0.603	0.375	0.572	0.7625	1.132	0.651	0.9836
HPAMAM-EGDE-3	0.781	0.280	0.529	0.8044	0.999	0.827	0.9878
HPAMAM-EGDE-4	0.848	0.256	0.361	0.8972	1.551	0.881	0.9953
HPAMAM-EGDE-5	0.646	0.326	0.373	0.8909	1.622	0.680	0.9941

#### Adsorption Isotherms

**FIGURE 6:**
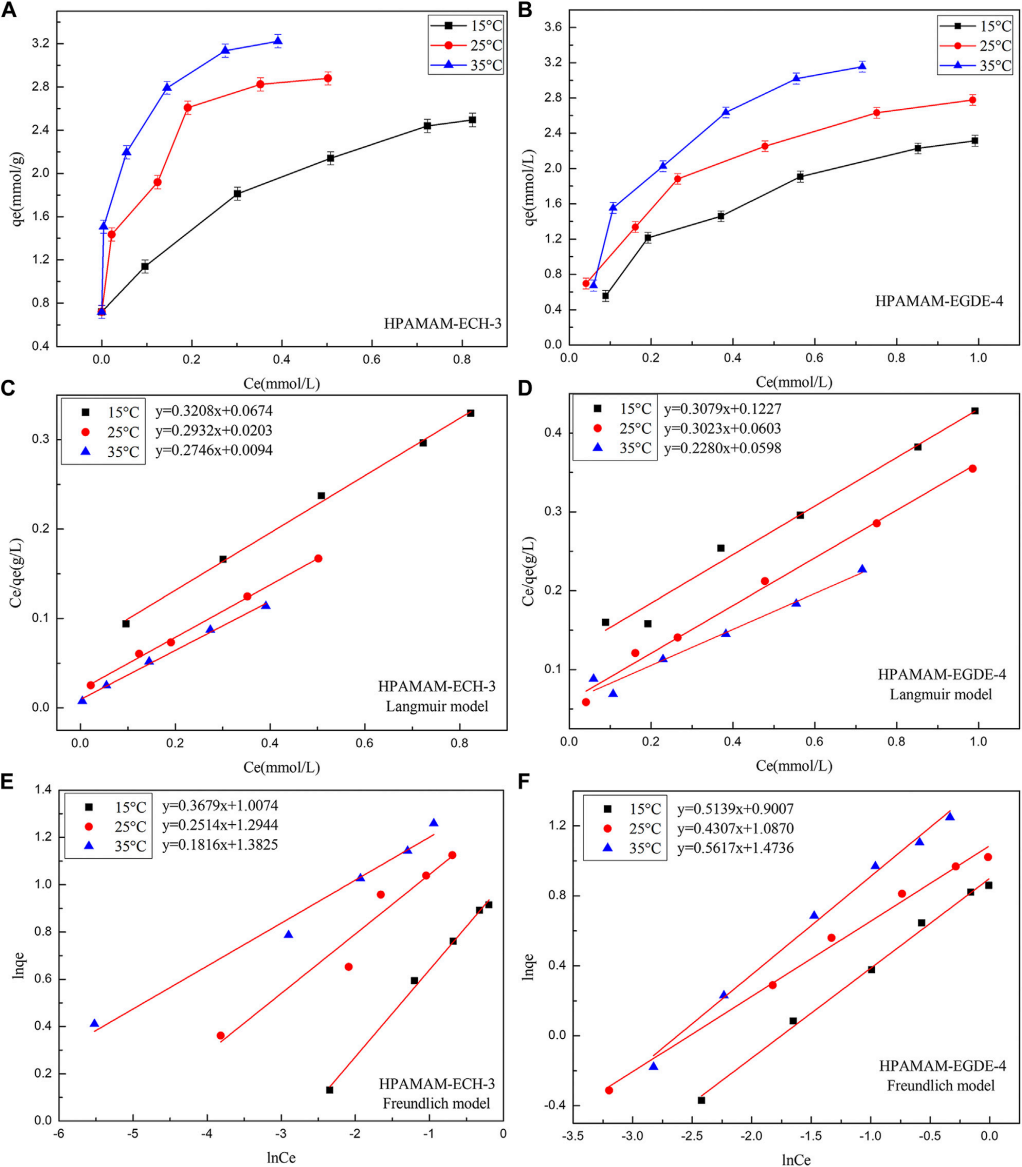
**(A**,**B)** The adsorbing capacities, **(C**,**D)** the Langmuir isotherms and **(E**,**F)** the Freundlich isotherms of HPAMAM-ECH-3 and HPAMAM-EGDE-4 at different initial concentrations and temperatures, pH 5.0 for Hg(II).

The adsorption isotherm of HPAMAM-ECH and HPAMAM-EGDE for Hg(II) were studied to depict the interactive behavior between adsorbent and adsorbate and the results were shown in Figure 6. With the increase of initial metal ion concentration, the adsorption increased accordingly due to the presence of higher driving force under high concentration. It was the high driving force that promoted the diffusion of metal ions and the adsorption capacity increased accordingly. When the initial metal ion concentration increased to a certain extent, the adsorption capacity would no longer increase on account of saturation of adsorbents. It can be also found that temperature had a remarkable effect on the adsorption capacity. It was worth mentioning that at lower initial concentrations (50 mg/L), HPAMAM-ECH-3 can completely adsorb Hg(II). The favorable of high temperature can be ascribed to the nature of the adsorption was endothermic.

The Langmuir and Freundlich equations were adopted to fit the experimental data. The Langmuir model assumes that adsorption occurs on a single layer of uniform surface with no interaction between the adsorbents, whereas the Freundlich model assumes heterogeneous adsorption with multiple molecular layers. The nonlinear expressions of the Langmuir and Freundlich models were as follows (Saadi et al., 2015):

$$\frac{C_{e}}{q_{e}}=\frac{C_{e}}{q_{max}}+\frac{1}{q_{max}K_{L}}$$ (5)

$$lnq_{e}=\;lnK_{F}\;+\frac{lnC_{e}}{n}$$ (6)

Where *q*_*e*_ (mmol/g) is the equilibrium adsorption capacity; *C*_*e*_ (mmol/L) is the Hg(II) concentration at adsorption equilibrium; *q*_*max*_ (mmol/g) is the maximum adsorption capacity; *K*_*L*_ is the Langmuir adsorption constant; and *K*_*F*_ and *n* are the Freundlich constants related to adsorption capacity and adsorption strength, respectively.

Figure 6 showed the fitting of the adsorption isotherms of HPAMAM-ECH-3 and HPAMAM-EGDE-4 at different temperatures to the Langmuir and Freundlich. For HPAMA-ECH-3 material, since the equilibrium concentration at low initial concentration was 0, the following five points were selected for fitting in order to ensure accuracy. From the correlation coefficients in Table 5, it can be concluded that the experiment data fitted Langmuir equation better than Freundlich equation for all cases of HPAMAM-ECH, revealing the adsorption of Hg(II) adsorption on HPAMAM-ECH obeyed the Langmuir adsorption isotherm. This implied that the adsorption of Hg(II) on HPAMAM-ECH followed the mechanism of monolayer adsorption (chemisorption) (Saadi et al., 2015). The difference was that for HPAMAM-EGDE samples, both Freundlich and Langmuir model had large correlation coefficients (*R* ^2^ > 0.96). This phenomenon indicated that monolayer adsorption and multi-layer adsorption occurred simultaneously in the adsorption process of HPAMAM-EGDE materials. The multi-layer adsorption of HPAMAM-EGDE samples may be related to the wrinkled structure on the surface of the material and the internal cavity structure.

**TABLE 5:** Langmuir and Freundlich isotherm constants for the adsorption of Hg(II) on HPAMAM-ECH-3 and HPAMAM-EGDE-4 at pH 5.0, 25°C.

T (°C)	Adsorbents	Langmuir			Freundlich		
		*q*_max_ (mmol/g)	*K*_*L*_	R^2^_L_	*K*_*F*_	*n*	R^2^_F_
15	HPAMAM-ECH-3	3.117	4.760	0.9970	2.738	2.718	0.9952
	HPAMAM-EGDE-4	3.248	2.509	0.9807	2.461	1.946	0.9948
25	HPAMAM-ECH-3	3.411	14.41	0.9969	3.649	3.978	0.9256
	HPAMAM-EGDE-4	3.308	5.013	0.9918	2.965	2.322	0.9920
35	HPAMAM-ECH-3	3.642	29.21	0.9946	3.985	5.507	0.9763
	HPAMAM-EGDE-4	4.386	3.813	0.9663	4.365	1.780	0.9887

Table 6 showed the comparison of the maximum adsorption capacity of the adsorbents prepared in this paper with those reported in other literatures. The adsorption capacity of HPAMAM-ECH samples and HPAMAM-EGDE samples exhibited competitive adsorption performance for Hg(II) than most adsorbents due to high density nitrogen and oxygen ligands. This was evidence that crosslinked HPAMAM adsorbents prepared in this way can be potentially used for capture of Hg(II) from wastewater.

**TABLE 6:** Comparison of adsorption capacity of different adsorbents for Hg(II) at 25°C.

Adsorbents	q_e_ (mmol/g)	References
Crosslinked HPAMAM with ECH	3.08	This study
Crosslinked HPAMAM with EGDE	2.78	This study
Polyamidoamine dendrimers functionalized magnetic graphene oxide	0.57	Ma et al. (2017)
PAMAM dendrimers modified attapulgite composites	1.00	Qin et al. (2019)
Sulfur-functionalized polyamidoamine dendrimer/magnetic Fe_3_O_4_	0.8	Luan et al. (2021)
Diethylenetriaminepentaacetic acid-modified cellulose	2.37	Li et al. (2019)
Thiol functionalized magnetic carbon nanotubes	0.86	Fan et al. (2019)
Schiff base functionalized magnetic Fe_3_O_4_	0.53	Zhao et al. (2020)
Amino- and thiol- polysilsesquioxane simultaneously coating on Poly (p-phenylenetherephthal amide) fibers	1.36	Wang et al. (2019)
Chitosan/amine modified diatomite composites	0.51	Fu et al. (2018)

#### Adsorption Selectivity

A series of binary ions coexistent systems, Hg(II)-Cu(II), Hg(II)-Co(II), Hg(II)-Cd(II), Hg(II)-Zn(II) and Hg(II)-Ni(II), were investigated to evaluate the adsorption selectivity of HPAMAM-ECH and HPAMAM-EGDE for Hg(II) and the results were listed in Table 7. It was obvious that HPAMAM-ECH and HPAMAM-EGDE exhibited excellent adsorption selectivity for Hg(II) from the binary metal ion systems, remarkably, the selective coefficients were infinite for Hg(II) in the presence of Co(II), Cd(II) and Zn(II). Therefore, the absorbents prepared in this work can probably be used for selective extracting and separating Hg(II) from aqueous system containing multiple metal ions.

**TABLE 7:** Adsorption selectivity of HPAMAM-ECH -3 toward Hg(II) at pH 5.0, 25°C.

System	Metal ions	q (mmol/g)	Selectivity coefficient (α)
Hg(II)-Cu(II)	Hg(II)	1.72	19.11
	Cu(II)	0.09	
Hg(II)-Co(II)	Hg(II)	1.31	∞
	Co(II)	0.00	
Hg(II)-Cd(II)	Hg(II)	1.84	∞
	Cd(II)	0.00	
Hg(II)-Zn(II)	Hg(II)	1.29	∞
	Zn(II)	0.00	
Hg(II)-Ni(II)	Hg(II)	1.98	15.23
	Ni(II)	0.13	

#### Desorption and Regeneration Performance

**FIGURE 7:**
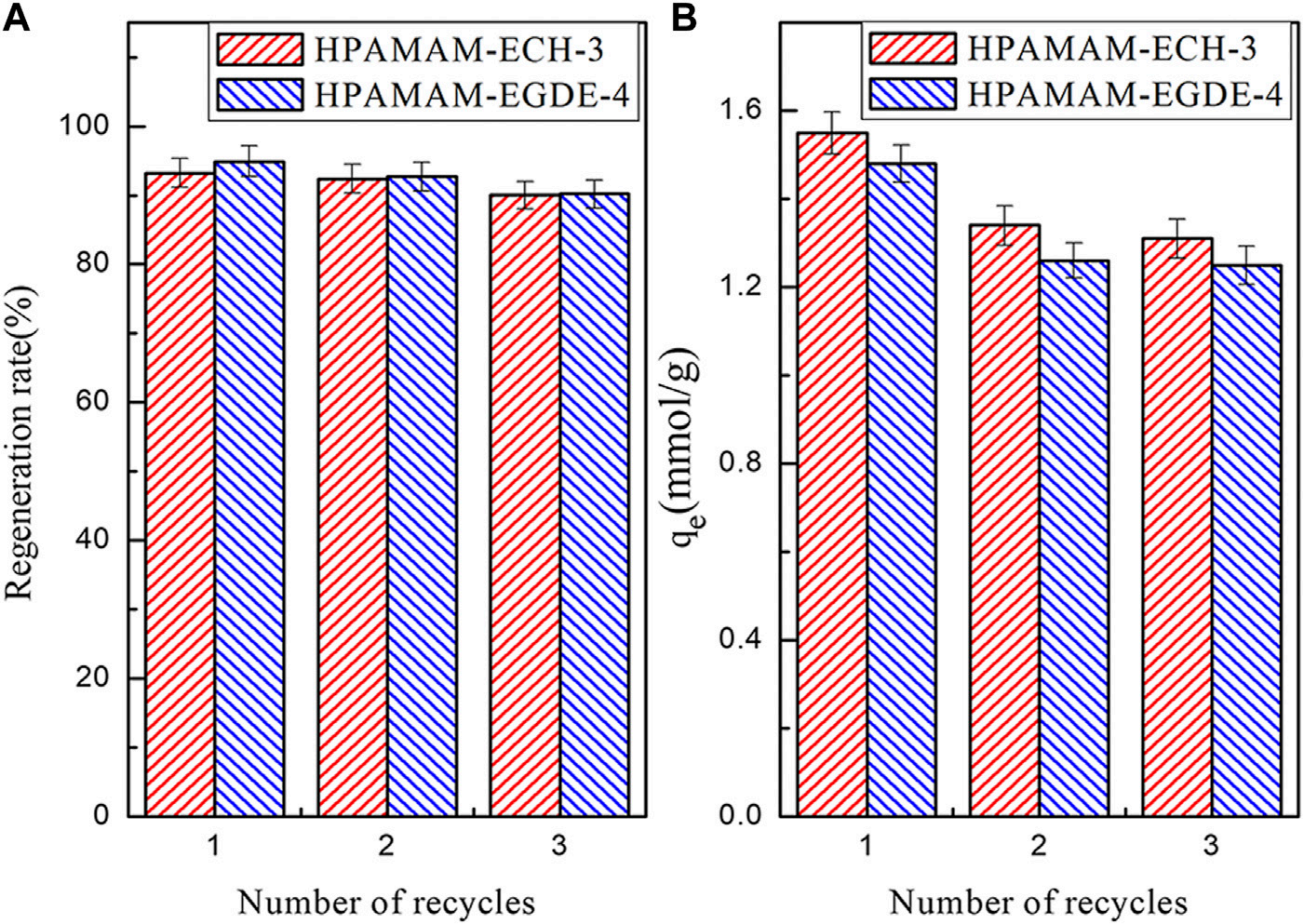
The regeneration rate **(A)** and elution regeneration **(B)** adsorption capacity of the adsorbents.

The regeneration rate and adsorption capacity of the HPAMAM-ECH-3 and HPAMAM-EGDE-4 after three elution-regeneration experiments were shown in the Figure 7. The regeneration rate (Figure 7A) of HPAMA-ECH-3 and HPAMA-EGDE-4 remained above 90% after three cycles. The adsorption capacity (Figure 7B) was still superior, which was 1.31 mmol/g for HPAMAM-ECH-3 and 1.25 mmol/g for HPAMAM-EGDE-4, respectively. Therefore, we had reason to believe that the crosslinked HPAMAM adsorbents thus prepared possessed great potential land economic benefit due to satisfactory adsorption capacity and stable regeneration performance.

#### Adsorption Mechanism

According to the data in Table 2, the amino and amide functional groups contents of HPAMAM-ECH-3 and HPAMAM-EGDE-4 were determined to be 7.39 and 6.13 (mmol/g). Thus, the coordination ratio of (NH_2_ +CONH) to Hg(II) of can be calculated to be 2.03:1 (HPAMAM-ECH-3) and 1.40:1 (HPAMAM-EGDE-4). It can be found that the functional group utilization rate of HPAMAM-ECH sample was greater than that of HPAMAM-EGDE sample. This may be because the steric hindrance of the samples crosslinked by ECH was larger, hindering the entry of metal ions, while the samples crosslinked by long-chain EGDE were relatively loose. At the same time, the HPAMAM-EGDE sample had a relatively large specific surface area, and there were wrinkles and cavities on the surface, which may lead to the simultaneous chemical adsorption and physical adsorption, and improved the utilization rate of its functional groups.

**FIGURE 8:**
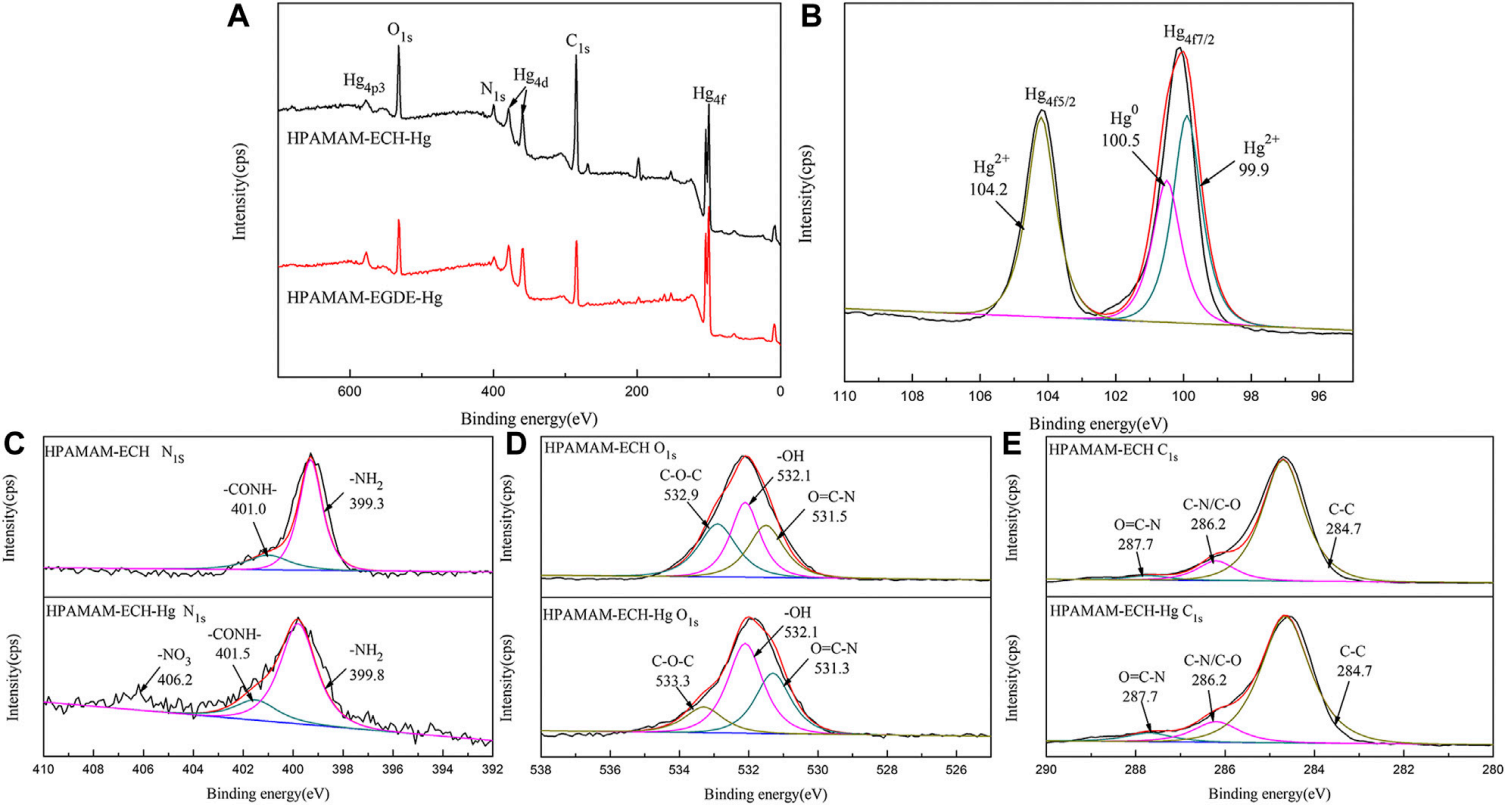
**(A)** Wide scan XPS spectra of HPAMAM-ECH-3 and high-resolution XPS spectra of **(B)** Hg_4f_, **(C)** N_1s_, **(D)** O_1s_, **(E)** C_1s_ of HPAMAM-ECH-3 before and after Hg(II) adsorption.

The XPS survey spectra of HPAMAM-ECH-3 after adsorption as well as the N_1s_, O_1s_ and C_1s_ high resolution spectra were used to gain further insight into the mechanism by comparing the changes of elements and binding energies before and after adsorption. The absorption peaks of Hg_4f_ and Hg_4d_ can be clearly seen in the Figure 8A, and the absorption peaks of Hg_4p_ can also be found, which verified the successful uptake of Hg(II). It can be observed from the high-resolution XPS spectra of Hg_4f_ (Figure 8B) that two absorption peaks located near 99.8 and 103.6 eV respectively corresponded to Hg_4f__5/2_ and Hg_4f 7/2_. While, the main peak of Hg_4f 7/2_ contained two peaks of Hg^2+^ and Hg^0^, which were located at 99.6 and 100.5 eV respectively, indicating that Hg(II) may have undergone a redox reaction (Xu et al., 2021). Correspondingly, Figure 8C showed that after adsorption of Hg(II), the binding energy shifted from 399.3 to 399.8 eV for –NH_2_ and from 401.0 to 401.5 eV for CONH due to the electrostatic interaction, and the peak areas were also increased. In addition, there was also a small peak near 406.2 eV in the spectrum, which belonged to the binding energy peak of N element in -NO_3_. For O_1s_ (Figure 8D), the peak of CONH moved from 531.5 to 531.3 eV, while that of C-O-C group moved from 532.9 to 533.3 eV. After adsorption of Hg(II), the high-resolution C_1s_ spectra (Figure 8E) almost didn’t change, indicating the existence form of C element remained the same. From the XPS analysis, we can conclude that the N in the amino group and amide group, and O in the C-O-C and amide group in the materials were involved in Hg(II) adsorption and the main adsorption mechanism for Hg(II) was complexation.

## Conclusion

Two kinds of HPAMAM adsorption materials were successfully prepared for efficient capture of Hg(II) by cross-linking HPAMAM with ECH and EGDE, respectively. Materials crosslinked with EGDE were structurally looser than those crosslinked with ECH because EGDE had longer molecular chains. The influence of the amount of crosslinking agent on the adsorption capacity followed a descending order: HPAMAM-ECH-3 > HPAMAM-ECH-4 > HPAMAM-ECH-5 > HPAMAM-ECH-2 for HPAMAM-ECH samples and HPAMAM-EGDE-4 > HPAMAM-EGDE-3 > HPAMAM-EGDE-5 > HPAMAM-EGDE-2 for HPAMAM-EGDE samples. The optimum adsorption pH was 5.0 and the adsorption kinetics followed pseudo-second-order kinetic model. Adsorption thermodynamics showed that HPAMAM-ECH samples were monomolecular adsorption, while HPAMAM-EGDE samples were a combination of monomolecular adsorption and multi-layer adsorption. Both materials had demonstrated excellent selectivity and regeneration properties.

## Data Availability

The original contributions presented in the study are included in the article/Supplementary Material, further inquiries can be directed to the corresponding authors.
